# Clinical Management of Nonsteroidal Anti-inflammatory Drug Hypersensitivity

**DOI:** 10.1097/WOX.0b013e3181625db2

**Published:** 2008-02-15

**Authors:** Mario Sánchez-Borges

**Affiliations:** 1 The Allergy and Immunology Department, Centro Médico-Docente La Trinidad and Clínica El Avila, Caracas, Venezuela

**Keywords:** aspirin, drug hypersensitivity, nonsteroidal anti-inflammatory drugs, NSAIDs

## Abstract

Hypersensitivity diseases caused by nonsteroidal anti-inflammatory agents are relatively common in the population. This article summarizes the present understanding on the various allergic and nonallergic clinical pictures produced through hypersensitivity to these drugs using the pathogenic classification of hypersensitivity reactions recently proposed by the Nomenclature Committee of the World Allergy Organization to guide clinicians in the diagnosis and management of patients with these conditions.

## 

A large proportion of the population is exposed to nonsteroidal anti-inflammatory drugs (NSAIDs) worldwide from either medical prescription or self-medicated [1]. It is then not surprising that these drugs constitute the second major cause of hypersensitivity reactions to drugs after β-lactamic antibiotics.

The prevalence of these reactions in the population varies between 0.1% and 0.3%, [2] and therefore, it is very important for clinicians to recognize and properly treat patients suffering from NSAID hypersensitivity. This article reviews the information presently available on the clinical manifestations, diagnosis, and management of these reactions.

## Nonsteroidal Anti-inflammatory Drugs

Pharmacology textbooks define NSAIDs as compounds that antagonize inflammation through the inhibition of a group of enzymes known as cyclooxygenases (COXs) [3]. Some drugs, notably pyrazolones and acetaminophen, were previously not classified into this group because they did not inhibit COX enzymes. In recent years, new COX isoenzymes have been described, such as COX-2b and COX-3, that can be selectively antagonized by these drugs, and therefore they would fit into the NSAID category [4,5].

Classic NSAIDs that inhibit both major COX isoen-zymes, COX-1 and COX-2, can be classified according to their chemical structure as depicted in Table 1. A second classification is based on the selectivity of NSAIDs for inhibition of COX isoenzymes (Table 2).

**Table 1:** Chemical Classification of NSAIDs

Chemical Group	Drugs
Alkanones	Nabumetone
Anthranilic acids (fenamates)	Meclofenamic acid, mefenamic acid
Arylpropionic acids	Fenoprofen, flurbiprofen, ibuprofen, ketoprofen, naproxen, oxaprozin
Enolic acids	Oxicams (piroxicam, tenoxicam), pyrazolidinediones (oxyphenthatrazone, phenylbutazone)
Heteroaryl acetic acids	Diclofenac, ketorolac, tolmetin
Indole and indene acetic acids	Etodolac, indomethacin, sulindac
Para-aminophenol derivatives	Acetaminophen (paracetamol)
Pyrazol derivatives	Aminopyrine, antipyrine, dipyrone
Salicylic acid derivatives	Aspirin, choline magnesium trisalicylate, diflunisal, olsalazine, salicylsalicylic acid, salsalate, sodium salicylate, sulfasalazine

**Table 2:** Classification of NSAIDs According to Their Selectivity for COXs

Selectivity	Drugs
Weak COX inhibitors	Acetaminophen, salsalate, salicylamide, sodium salicylate, choline-magnesium trisalicylate
COX-1/COX-2 inhibitors	Piroxicam, indomethacin, sulindac, tolmetin, ibuprofen, naproxen, fenoprofen, meclofenamate, mefenamic acid, diflunisal, ketoprofen, diclofenac, ketorolac, etodolac, nabumetone, oxaprozin, flurbiprofen
COX-2 preferential inhibitors	Nimesulide, meloxicam
COX-2 selective inhibitors	Celecoxib, rofecoxib, valdecoxib, parecoxib, etoricoxib, lumiracoxib

## Clinical Spectrum and Pathogenesis

A wide variety of clinical manifestations can be produced by NSAIDs. Using the classification proposed by the Nomenclature Committee of the World Allergy Organization, [6] the following types of hypersensitivity reactions can be considered:

### Allergic Hypersensitivity

Immunologic reactions to NSAIDs can be subdivided into immediate (mediated by immunoglobulin E [IgE]) and delayed (mediated by lymphocytes).

#### Immediate Reactions

##### Urticaria and Angioedema

Immunoglobulin E-mediated cutaneous reactions have been described for pyrazolones, [7] acetaminophen, [8] and aspirin [9].

##### Allergic Anaphylaxis

Reported for ibuprofen, [10] ketorolac, [11] indomethacin, sulindac, zomepirac, [12] fenoprofen, meclofenamate, naproxen, piroxicam, tolmetin, [13] glafenine, acetaminophen, aspirin, diclofenac, and celecoxib [14].

#### Delayed Reactions

These include cell (T lymphocyte)-mediated type IV hypersensitivity reactions involving specific organs and systems.

##### Skin Diseases

###### Fixed-drug Eruptions

Characterized by erythematous plaques recurring in the same anatomical site in every occasion the drug is administered. Metamizole, piroxicam, phenylbutazone, paracetamol, aspirin, mefenamic acid, diclofenac, indomethacin, ibuprofen, diflunisal, naproxen, and nimesulide have been incriminated [15].

###### Toxic Epidermal Necrolysis, Stevens-Johnson Syndrome, and Acute Generalized Exanthyematous Pustulosis (AGEP)

These serious skin reactions belong to the erythema multiforme spectrum of bullous eruptions and can be associated with NSAIDs [16].

Stevens-Johnson syndrome (SJS) is a severe diffuse mucocutaneous eruption causing erythematous or purpuric macules, blisters, or target lesions with no more than 10% skin detachment, accompanied by systemic manifestations, occurring 1 to 8 weeks after administration of incriminated medications [17]. Toxic epidermal necrolysis (TEN) involves 30% or more skin detachment, whereas between 10% and 30% detachment is applied to the term SJS-TEN overlap syndrome.

Among NSAIDs, oxicams, phenylbutazone, and oxyphenbutazone have been responsible more often [16,18]. Recently, a great deal of attention has been given to the association of SJS/TEN with the use of new COX-2 inhibitors, especially valdecoxib and celecoxib [19-21].

Acute generalized exanthematous pustulosis is a rare condition characterized by a rapid-onset pustular eruption involving most of the body. Typical lesions are generalized, nonfollicular, pinhead-sized sterile pustules on an erythematous background that are associated with fever and neutrophilia [22]. Histopathologic features include papillary edema, a mixed upper dermal perivascular infiltrate, and a spongiform subcorneal pustule. Activated HLA-DR-positive CD4 and CD8 T cells, interleukin-8, interleukin-5, and granulocyte-macrophage colony-stimulating factor are present in the tissue. The NSAIDs associated with acute generalized exanthematous pustulosis more often are ibuprofen, phenylbutazone, naproxen, acetylsalicylic acid, valdecoxib, and celecoxib.

###### Contact and Photocontact Dermatitis

Contact with NSAIDs can induce itchy, erythematous, edematous, and vesicular lesions, and photocontact dermatitis, an exaggerated or abnormal cutaneous response to light. Among NSAIDs, diclofenac, indomethacin, flurbiprofen, bufexamac, etofenamate, flufenamic acid, ibuprofen, ketoprofen, and tiaprofenic acid are the most common inducers of contact dermatitis. Cross-reactivity between some chemically related NSAIDs has been observed [23].

###### Maculopapular Eruptions

Virtually all NSAIDs are able to produce maculopapular eruptions, one of the most common cutaneous adverse effects of NSAIDs. Ibuprofen, pyrazolones, flurbiprofen, diclofenac, and celecoxib have been more frequently involved.

##### Pneumonitis

Some NSAIDs such as aspirin, sulindac, ibuprofen, and naproxen can induce allergic pneumonitis. The NSAID-induced pneumonitis can be suspected from a temporal relationship between lung infiltrates and drug administration [24]. Most patients will improve after drug discontinuation, although corticosteroids may be needed for severe or persistent cases.

##### Aseptic Meningitis

The NSAIDs are the medications more often involved in the production of drug-induced meningitis. Clinical features include fever, headache, photophobia, neck stiffness, nausea, vomiting, arthralgia, myalgia, rash, and abdominal pain [25]. Ibuprofen, sulindac, naproxen, tolmetin, diclofenac, ketoprofen, piroxicam, indomethacin, rofecoxib, and celecoxib have been associated with aseptic meningitis. Casas-Rodriguez et al [26] observed that 61% of ibuprofen-related meningitis occurred in patients with connective tissue diseases, mainly systemic lupus erythematosus. Management includes drug withdrawal, systemic corticosteroids, and avoidance of re-exposure to drugs from the same family as the causal drug.

##### Nephritis

Rarely, in aged patients with normal kidneys, NSAIDs may trigger a spectrum of nephritides ("NSAID nephropathy"), including tubular, interstitial, acute or subacute tubulointerstitial nephritis, chronic interstitial nephritis with papillary necrosis, and tubulointerstitial nephritis combined with nephrotic syndrome. The NSAIDs may also produce glomerulopathies such as minimal change nephropathy, membranous glomerulonephritis, and focal sclerosis [27,28].

##### Hepatitis

Rarely, NSAIDs, among them niflumic acid, tolfenamic acid, diclofenac, fenoprofen, ibuprofen, indomethacin, naproxen, piroxicam, pirprofen, and sulindac, induce allergic hepatitis that can be mixed, cytolytic, or cholestatic. It is observed in elderly women taking multiple medications [29].

Herdeg et al [30] reported a case of metamizole-induced allergic cholestatic hepatitis characterized by generalized exanthema and increased liver enzymes. Sensitization to the drug was confirmed by means of lymphocyte transformation test.

### Nonallergic Hypersensitivity

Composed of manifestations at the respiratory tract and skin, and nonallergic anaphylaxis.

#### Respiratory Hypersensitivity

Aspirin-induced asthma, aspirin-intolerant asthma, or aspirin-exacerbated respiratory disease (AERD) is characterized by asthma, rhinosinusitis, nasal polyposis, and aspirin/NSAID hypersensitivity. Asthmatic reactions induced by NSAIDs occur in 5% to 20% of adult asthmatic patients [31]. The pathogenesis seems to involve the combined effects of chronic inflammation and a pharmacogenetic abnormality of arachidonic acid metabolism in response to NSAIDs. This leads to sulfidoleukotriene overproduction and to a decrease in anti-inflammatory prostaglandin E2 from insufficient COX-2 activation, leading to additional leukotriene synthesis [32,33]. Excellent reviews on this topic have been recently published [34].

#### Cutaneous Hypersensitivity

**Figure 1:**
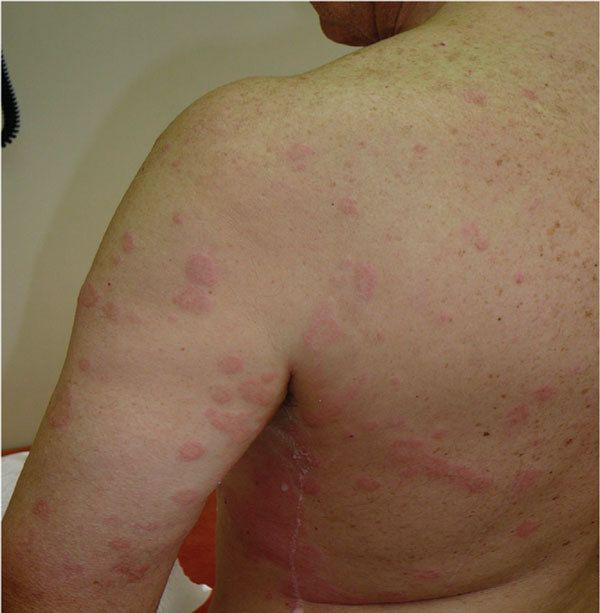
**A 62-year-old man with CIU exacerbated by the ingestion of sodium diclofenac**.

The cutaneous pattern of NSAID-induced cross-reactions includes cross-reacting urticaria and angioedema in patients with or without chronic idiopathic urticaria (CIU) (Figure 1). The mechanisms are not completely understood, but in patients with CIU, COX-1 inhibition has been demonstrated [35,36].

#### Nonallergic Anaphylaxis

Previously known as anaphylactoid or pseudoallergic reaction, it is observed in cross-reactive patients and presumably mediated by inhibition of COX-1 [37].

## Diagnostic Methodology for Adverse Reactions Induced by NSAIDS

The choice of diagnostic tests is based on the clinical picture and possible pathogenesis.

### Reactions Mediated by IgE

Although intradermal injection of pyrazolones has been proposed for diagnostic purposes, no correlation with the clinical picture was observed [7]. Presently, no standardized reagents for immediate-type skin tests with NSAIDs are available.

### Delayed-type Reactions

Patch tests constitute a simple, fast, and relatively safe method for the diagnosis of delayed reactions to NSAIDs. For fixed-drug eruptions, lesional skin should be used for the test [38-40]. When photoallergy is suspected, photopatch tests are indicated [41]. Concentrations of NSAIDs commonly used for patch tests are summarized in Table 3.

**Table 3:** Concentrations of NSAIDs for Patch Testing


**Drug**	Concentration (%)*

Acetylsalicylic acid	5
Bufexamac	2-5
Celecoxib	10
Diclofenac	0.1-2
Etofenamate	5
Fenoprofen	1-5
Flufenamic acid	5
Flurbiprofen	1-5
Ibuprofen	1-5
Ibuproxan	5
Indomethacin	1-5
Ketoprofen	1-10
Metamizole	10-50
Meloxicam	1
Naproxen	2-10
Nimesulide	5-10
Oxyphenbutazone	1-10
Paracetamol	5
Phenylbutazone	1-10
Piroxicam	0.5-1
Salicylic acid	1
Tenoxicam	0.5-1
Tiaprofenic acid	1-5
Valdecoxib	1-10

*In white petrolatum.

It must be noticed that in some cases, a rechallenge with the drug is not recommended because of the high risk of severe and generalized reactions [42]. Intradermal and scratch tests with reading at 48 and 72 hours have also been used to confirm delayed hypersensitivity to NSAIDs [43].

The lymphocyte transformation test measures the in vitro proliferative response of T cells stimulated by the drug. Although this test is worth to be considered, it is only available in some laboratory facilities in specialized centers [44].

### Nonallergic Reactions

For respiratory and cutaneous cross-reactions, the criterion standard continues to be the controlled oral provocation test carried out in the appropriate medical facilities by physicians experienced in this kind of test and with easy access to medications and equipments necessary for the treatment of reactions [45,46]. Bronchial and nasal inhalation challenges with aerosols of L-lysine acetylsalicylic acid have also been used [47,48]. An algorithm for the diagnosis and management of patients with cutaneous NSAID reactions has been proposed [46].

De Weck et al [49] have developed 2 in vitro assays done with blood basophils: the leukotriene release test and the basophil activation test. These tests require, however, special equipments and reagents, including a flow cytometer, and therefore are more expensive and limited to some centers [50].

## Patient Management

### IgE-mediated Reactions

Patients reacting to a single NSAID can receive NSAIDs from a different chemical group (see Table 1). In general, it is recommended not to use in these patients NSAIDs from the same group because cross-reactions between NSAIDs of similar chemical structure occur.

### Delayed-type Reactions

Discontinuation of offender medication and pharmacological treatment with corticosteroids and antihistamines are recommended.

Patients with severe reactions of the TEN/SJS type should be transferred to intensive care or burn units. Intravenous immunoglobulins have been used in patients with TEN, based on its content of natural anti-Fas antibodies, and a reduction of mortality has been shown in some studies [51]. Systemic therapy with infliximab (anti-TNF-α) induced rapid improvement of skin lesions in patients with TEN [52].

### Nonallergic Reactions

#### Aspirin-exacerbated Respiratory Disease

The following measures are recommended for patients with AERD:

• Avoidance of all classic COX-1 inhibitors.

• Pharmacological treatment with topical and systemic corticosteroids, leukotriene receptor antagonists, and 5-lipoxygenase inhibitors, antibacterials, and antifungals [53].

• Use of alternative NSAIDs (acetaminophen, salsalate, dextropropoxyphene, opioids, ergotamine, hyoscine, sodium salicylate, salicylamide, choline-magnesium trisalicylate, floctafenine). Acetaminophen and other weak inhibitors of COX-1 are generally well tolerated by these patients at lower doses, but if the dose is increased, respiratory reactions can occur [54].

• Specific COX-2 inhibitors are tolerated by most patients with AERD [55].

• Desensitization is indicated for selected sensitive patients who need to receive NSAIDs for other medical conditions and for patients with severe corticosteroid-dependent AERD [56].

#### Cutaneous Reactions and Nonallergic Anaphylaxis

Patients with cross-reacting urticaria/angioedema and nonallergic anaphylaxis should be managed as follows:

• Avoidance of COX-1 inhibitors.

• Alternative medications as mentioned above (see aspirin-exacerbated respiratory disease).

• COX-2 inhibitors are safe for most of these patients, but long-term use of coxibs is not recommended because of the cardiovascular risks associated with them. In such cases, preferential inhibitors of COX-2 may be helpful [57,58].

• Desensitization is generally not recommended.
